# Reasonable suspicion in reporting child maltreatment: a survey among German healthcare professionals

**DOI:** 10.1186/s13034-021-00381-7

**Published:** 2021-06-14

**Authors:** Oliver Berthold, Andreas Jud, Marion Jarczok, Jörg M. Fegert, Vera Clemens

**Affiliations:** 1 Department of Child and Adolescent Psychiatry/Psychotherapy, University of Ulm, Steinhövelstr. 5, 89073 Ulm, Germany; 2 Sciences and Arts, School of Social Work, Lucerne University of Applied, Lucerne, Switzerland; 3 Child Abuse Clinic, Department of Pediatrics, DRK Kliniken Berlin | Westend, Spandauer Damm 130, 14050 Berlin, Germany

**Keywords:** Child protection, Child maltreatment, Reasonable suspicion, Healthcare professionals, Health care system, Child protection system

## Abstract

**Background:**

With regular contacts to the general child population, healthcare professionals could play an important role in the detection of child maltreatment. However, a majority of child maltreatment cases go unnoticed by the healthcare system. Child protection legislations usually offer terms like “reasonable suspicion” to corner a threshold that warrants reporting to child protection services (CPS) is defined as. The indistinct legal terminology leads to marked differences in the interpretation of this threshold. Therefore, we aimed to systematically assess the understanding of reasonable suspicion and subsequent handling of cases in the German context.

**Methods:**

A cross-sectional online survey was conducted among 2485 physicians and psychotherapists working with children and adolescents. Field access was gained by German professional associations. Via case vignettes, predictors of thresholds for reporting were assessed.

**Results:**

The probability of a report to CPS increased positively with the degree of suspicion for maltreatment. However, even if participants were certain that child maltreatment occurred, 20% did not chose to report to CPS. Training in child protection lowered the professionals’ threshold for reasonable suspicion; experience with child protection cases and good knowledge of the legal framework increased the likelihood to report an alleged situation of child maltreatment to CPS.

**Conclusion:**

Our data show that a significant proportion of health care professionals are uncertain about estimating reasonable suspicion and on how to proceed when there are strong indications for child maltreatment Therefore, data point towards the relevance of training in child protection among healthcare professionals in order to improve detection and adequate handling of cases of child maltreatment.

**Supplementary Information:**

The online version contains supplementary material available at 10.1186/s13034-021-00381-7.

## Introduction

With regular contacts to the general child population, healthcare professionals play an important role in the detection of child maltreatment [1]. However, to strengthen families and protect a maltreated child from further harm, healthcare resources are often insufficient. Moreover, they lack a public mandate to intervene if parents are unwilling or not able to cooperate appropriately with healthcare professionals. Mandatory interventions are reserved for public child protection, its organizations are usually labelled child protective services (for a framework of mapping child protection agencies, see Trocmé, Akesson and Jud, 2016). Most countries’ legal frameworks weigh the medical professional privilege against the right or even a mandate to report alleged incidents of child maltreatment to child protective services (CPS) [2]. Whether legislation opted for mandatory reporting or not, usually there is a threshold defined when the passing of personal data to child protection services (CPS) is warranted. In the U.S., the threshold that warrants mandatory reporting to CPS is defined as “reasonable suspicion”. Levi et al. found remarkable differences in the interpretation of this threshold among U.S. pediatricians [3]. The German legal threshold of “gewichtige Anhaltspunkte” as defined by the Federal Child Protection Act (German: “Bundeskinderschutzgesetz”) roughly translates to reasonable suspicion. These concepts are, however, indeterminate legal terms, i.e. it is not operationalized what this threshold means in a specific case. Consequently, the legal threshold is largely at the discretion of individual healthcare professional and will likely not only depend on child and family needs, but factors associated with the decision-maker (e.g. professional experience) or the organization [4]. For many healthcare professionals, the margin of discretion is rather a source of insecurity [5, 6]. Literature underlines that healthcare professionals see reporting as challenge [7, 8]. This is alarming as medical professionals in doubt of the right decision might “play it safe” and decide against involving CPS in cases of alleged child maltreatment—which, consequently, might deprive the child of adequate support and protection.

Child maltreatment is highly prevalent. A retrospective survey on the prevalence of child maltreatment revealed a percentage of 31% of N = 2510 participants victimized by any type of at least moderate child maltreatment in Germany [9]. However, reporting rates are low in Germany. CPS assessed at total of 1,36,925 alleged incidents of child maltreatment in Germany in 2018 [10]. The number transfers to a rate of 12 children per 1000 child residents. A total of 50,412 cases have been substantiated in 2018 or 3.8 per 1000 child residents. Importantly, only 9580 alleged incidents (7%) had been reported by the health care system. In comparison, 6.6 million children are referred to child protection agencies in the U.S. each year [11]. This figure is 48 fold higher in a country with only about 4times as many residents compared to Germany. The World Health Organization estimates that up to 90% of child maltreatment cases in Europe are not properly addressed in the health system [1]. Adequate rates of reporting by the healthcare system are particularly important for early childhood when children are not yet seen by other institutions, such as schools.

We need to better understand the reasons why healthcare professionals do or don’t report child abuse. Only then we can develop strategies to increase awareness and willingness to intervene in cases of suspected maltreatment. However, so far no systematic evidence exists to understand how healthcare professionals in Germany understand and apply the legal framework in child protection cases. Therefore, we conducted a nationwide survey among child healthcare professionals in Germany.

## Methods

### Sample

The target group of the survey was defined as pediatricians, child and adolescent psychiatrists, pediatric surgeons as well as child psychotherapists. These populations were accessed via professional associations (see Table 1). All medical associations and 11 out of 12 psychotherapeutic associations agreed to participate. Members of the participating associations who had agreed to receive emails by the associations were contacted to complete the online survey via at least two separate e-mails or newsletters or via the association’s website. As an incentive to participate, tickets for the Congress of the German Association for Child and Adolescent Psychiatry, Psychosomatics and Psychotherapy and the Congress of the German Society of Pediatrics and Adolescent Medicine were raffled. Overall, 2487 health care professionals took part, including 1842 physicians and 645 (psychological) psychotherapists. The age and gender distribution of the various groups surveyed is shown in Table 1. The distribution of physician participation across the various federal states roughly corresponds to the size of the federal states with the highest participation in North Rhine-Westphalia (433 participating physicians), followed by Bavaria (291) and Baden-Württemberg (267). The response among psychotherapists was different for the individual federal states, also due to different participation of the respective chambers. Bavaria is in first place with 202 psychotherapists participating, followed by Lower Saxony (73) and Baden-Württemberg (47) (response rate across all participants: 3.92%, for details see Additional file 1).

**Table 1:** Sample characteristics

		Medicine				Psychological psychotherapists			Chi^2^/p-value
		Pediatricians	Pediatric surgeons	Child and adolescent psychiatrists	Medicine total	Child and adolescent psychotherapists	Adult psychotherapists	Psychological psychotherapists total	
Participants	n	1581	58	203	1842	397	248	643	
Gender^a^	Female (%)	68	43	61	66	81	80	81	68.96/< 0.001
Age	< 41 (%)	42	21	30	40	34	34	34	
	41–60 (%)	47	60	61	49	57	47	53	
	> 60 (%)	11	19	9	11	10	19	13	44.99/< 0.001
Place of Work	> 20.000 Inhabitants (%)	82	98	82	82	68	71	69	57.20/< 0.001
Part of an interdisciplinary child protection team	Member (%)	13	60	16	15	6	7	6	146.77/< 0.001
Ever had training in child protection	(%)	29	59	29	30	20	22	21	43.27/< 0.001
At least 1 child protection case during the case 12 months (%)	(%)	80	95	91	81	74	45	63	184.27/< 0.001

^a^The 11 respondents who did not classify themselves as male or female are not considered as a separate group in the statistical models

### Measures

Demographic questions comprised gender, age group, profession, whether the participants ever attended a child protection specific training (yes/no), whether they are part of an interdisciplinary child protection team (child protection group, outpatient clinic or similar, answer: yes/no), in how many child protection cases the participants were involved with in the last 12 months and in which state the participants work professionally.

The participants were first asked about their knowledge of the Federal Child Protection Act.

Then case vignettes were presented. Pediatricians and pediatric surgeons were shown a case vignette with a physical injury suspicious for physical abuse (somatic case vignette), child and adolescent psychiatrists and psychotherapists a case vignette with the suspicion for neglect (psychiatric case vignette). In the two different vignettes we addressed specific knowledge of somatic and psychological aspects of maltreatment – therefore each group received only the vignette for their field of expertise. The case report in the pediatric vignette is a translation and adaptation of a vignette earlier published by Lee and colleagues [12] and describes the case of a premobile infant with a distal femur fracture; literature suggest for this case a probability of physical abuse at about 40% [13]. In addition, no medical history was available in the current pediatric vignette and the infant was presented to the medical system with considerable delay. Both aspects further increase the probability of child maltreatment. The mental health professionals’ vignette describes a 3 and 7 year old sibling couple whose single mother comes to a doctor’s appointment under the influence of alcohol with the children. In accordance with the “Hamburg catalogue”, a German assessment tool for CPS with comparably high thresholds, the vignette details a situation of reasonable suspicion for neglect [14]. In summary, the theoretical probability for child maltreatment in both vignettes is elevated and likely > 50%.

The participants were asked whether the respective cases offered reasonable suspicion for maltreatment (yes/no). Furthermore, the participants were asked about how likely this case will result in child maltreatment with the following options to respond: safely ruled out, no indication of child maltreatment, low probability, medium probability, high probability or child maltreatment substantiated. In the next step, participants were asked how they would handle the specific case based on their work experience and their knowledge of the legal framework. The possible options were: reporting to CPS; reporting to police, observation by a paediatrician in private practice and none of the above.

### Ethics/data protection

The study was reviewed and approved by the data protection officer of the University of Ulm. After consultation with the institutional review board of the University of Ulm, there is no requirement for an ethics vote due to the anonymous character of the survey.

### Data analysis

In addition to descriptive analyses, correlations of binary outcome variables on knowledge and hypothetical action were examined with multivariate logistic regressions.

## Results

### Participants

A total of 2487 participants completed the survey. For a detailed analysis of the participants by occupational group and relevant occupational specifications, see Table 1.

### Association between the degree of suspicion and course of action

The majority of participants saw medium to high degree of suspicion in the case vignettes (see Table 2). While only 22.7% of pediatricians and pediatric surgeons saw no evidence of maltreatment or estimated a low to medium degree of suspicion, this was the case for 33% of child and adolescent psychiatrists and 35.7% of psychotherapists (Chi^2^ = 131.85, p < 0.001).

**Table 2:** Degree of suspicion for child maltreatment and proposed handling based on the case vignettes

	Pediatricians/pediatric surgeons	Child and adolescent psychiatrists	Psychological psychotherapists	Chi^2^/p-value
Degree of suspicion for child maltreatment according to participants				
No evidence of child maltreatment or low probability	56 (3.4%)	10 (4.9 %)	40 (6.2 %)	
Medium probability	315 (19.3 %)	57 (28.1 %)	189 (29.5 %)	
High probability	1151 (70.5 %)	111 (54.7 %)	299 (46.7 %)	
Confirmed child maltreatment	110 (6.7 %)	25 (12.3 %)	112 (17.5 %)	131.85/<0.001
Correct handling of the case according to participants				
Reporting to CPS	1189 (73.2 %)	187 (92.1 %)	510 (80.1 %)	
Reporting to police	69 (4.2 %)	1 (0.5 %)	1 (0.2 %)	
Observation by a pediatrician in private practice	203 (12.5 %)	11 (5.4 %)	80 (12.6 %)	
None of the aforementioned	163 (10 %)	4 (2.0 %)	46 (7.2 %)	64.24/<0.001

Presented as N (%)

The vast majority of participants answered, they would have reported the case to CPS—with highest rates for child and adolescent psychiatrists (92.1%) and lowest for pediatricians and pediatric surgeons (73.2%) (Chi^2^ = 64.24, p < 0.001; see Table 2).

**Fig. 1:**
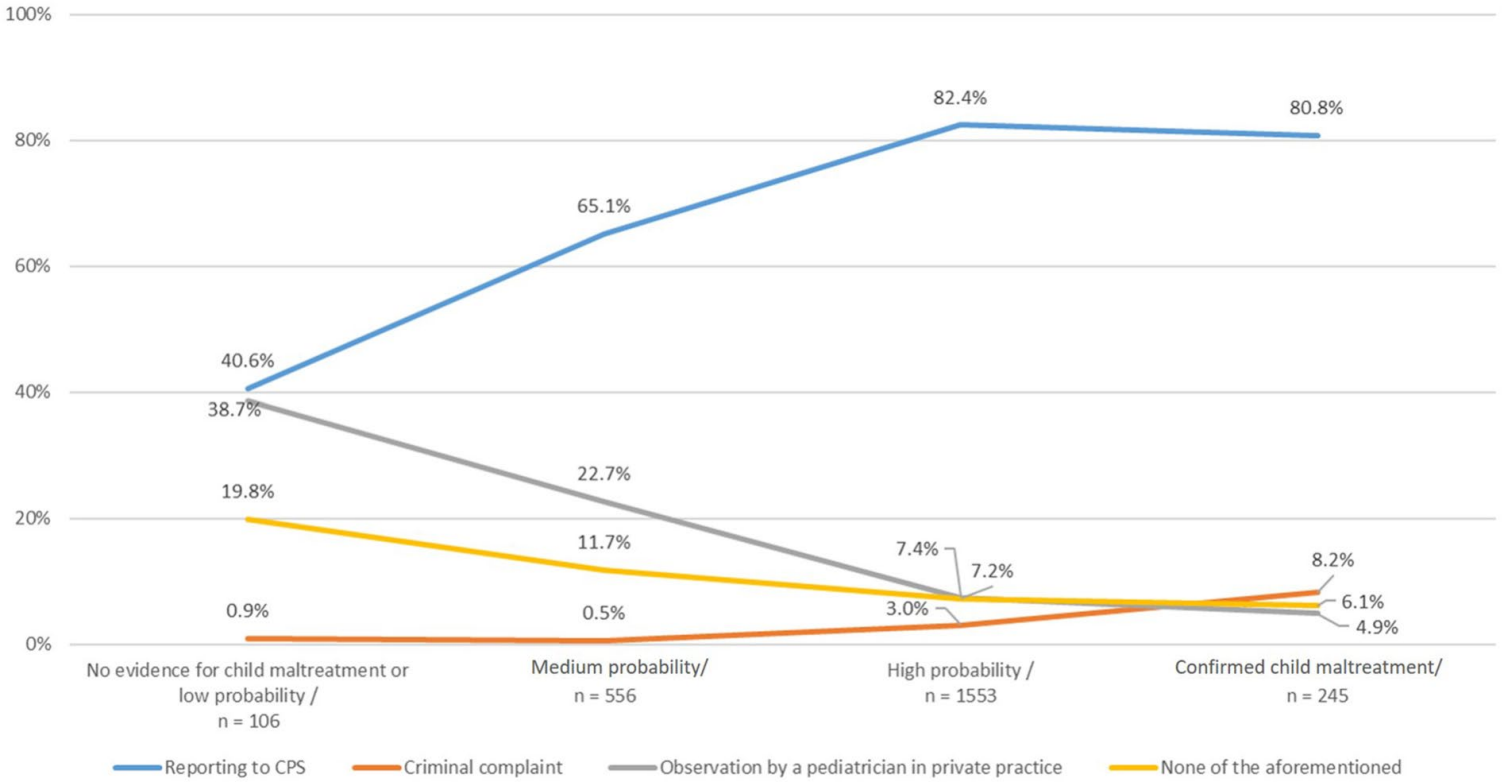
Capture: Association between the degree of suspicion and course of action. Legend: X-axis refers to the degree of suspicion, y-axis refers to the percentage of participants who prefer the respective course of action (in dependence of the degree of suspicion) N = 2460, Chi^2^ = 251.11, p < 0.001

Not surprisingly, the likelihood of a report to CPS increased with the degree of suspicion for maltreatment. The lower the threshold of suspicion was, the more likely the participants would have handled the case by “observation” only, i.e. with follow-up visits to the family physician/pediatrician without CPS involvement. The option to inform the police does not play a significant role in healthcare professionals’ decisions to handle situation of reasonable suspicion (see Fig. 1).

### Predictors for reasonable suspicion and CPS reporting

Among all participants, the threshold for reasonable suspicion for child maltreatment based on case vignettes was lowered if a child protection training course had been taken. Among child and adolescent psychiatrists and psychotherapists, female gender was another predictor for reasonable suspicion.

Pediatricians and pediatric surgeons had a higher chance to choose reporting to CPS based on the case vignettes if they had self-perceived good knowledge of German Federal Child Protection Act. Mental health professionals had a higher chance to report the case if they had at least one child protection case in the last 12 months and if they were psychiatrists compared to (psychologically trained) psychotherapists (see Tables 3, 4).

**Table 3:** Predictors for reasonable suspicion and reporting to CPS based on somatic case vignette (pediatricians and pediatric surgeons N = 1571)

	Reasonable suspicion				Reporting to CPS			
	Odds ratio^a^	z-Value	95% Confidence interval		Odds ratio^a^	z-Value	95% Confidence interval	
Female gender	1.17	0.59	0.696	1.960	1.08	0.58	0.839	1.380
Age above > 40 years	0.78	−0.92	0.464	1.320	0.93	−0.63	0.727	1.178
Urban place of work	1.11	0.36	0.618	2.008	1.07	0.50	0.808	1.431
1+ Child protection cases	1.55	1.62	0.911	2.643	1.23	1.51	0.940	1.618
Good knowledge of German Federal Child Protection Act	1.01	0.03	0.472	2.159	1.55	2.48*	1.097	2.188
Training in child protection	3.02	2.92**	1.437	6.350	1.14	0.94	0.868	1.493

*p < 0.05, **p < 0.01, ***p < 0.001

^a^An OR > 1 corresponds to a higher probability of assessing the situation as reasonable suspicion or informing CPS; comparison categories from top to bottom: male gender, age group < 41 years, rural place of work, 0 child protection cases, no or uncertain knowledge of the German Federal Child Protection Act , no child protection training

**Table 4:** Predictors for reasonable suspicion and reporting to CPS based on psychiatric case vignette (psychiatrists and psychotherapists N = 843)

Predictor	Reasonable suspicion				Reporting to CPS			
	Odds ratio^a^	z-Value	95% Confidence interval		Odds ratio^a^	z-Value	95% Confidence interval	
Female gender	1.73	2.25*	1.072	2.776	0.82	− 0.85	0.510	1.305
Age above > 40 years	1.09	0.40	0.705	1.696	0.77	− 1.28	0.516	1.150
Urban place of work	0.86	− 0.62	0.540	1.379	1.29	1.27	0.871	1.899
Studied medicine	1.54	1.54	0.889	2.683	2.42	3.04**	1.370	4.285
1+ Child protection cases	0.95	− 0.24	0.601	1.487	1.62	2.43*	1.098	2.387
Good knowledge of German Federal Child Protection Act	1.59	1.38	0.821	3.094	0.92	− 0.30	0.539	1.577
Training in child protection	2.54	2.66**	1.278	5.054	1.69	1.93	0.991	2.877

*p < 0.05, **p < 0.01, ***p < 0.001

^a^An OR > 1 corresponds to a higher probability of assessing the situation as reasonable suspicion or informing CPS; comparison categories from top to bottom: male gender, age group < 41 years, rural place of work, 0 child protection cases, no or uncertain knowledge of the German Federal Child Protection Act , no child protection training

## Discussion

This is the first analysis assessing systematically the understanding of “reasonable suspicion” and the application of the legal framework in specific child protection cases in healthcare professionals in Germany—and one of few similar analyses worldwide.

The overwhelming majority of respondents saw at least average probability for child maltreatment in the case vignettes. A percentage of 23 to 36% (depending on the profession) of participants however had no or low suspicion for child maltreatment. This finding suggests high heterogeneity and uncertainty in the assessment of particular cases—a fact well established in the empirical literature for all professionals in child protection [15, 16]. Empirically validated key risk factors for child maltreatment have been used in both case vignettes, which in turn suggests insufficient knowledge on the risk factors of child maltreatment. In consequence, child protection training increased the likelihood of perceiving the situation in the vignette as reasonable suspicion for child maltreatment.

Literature underlines insufficient knowledge of child maltreatment in healthcare professionals in Germany: in a previous study assessing reporting behavior of pediatricians and child and adolescent psychiatrists in the German capital Berlin, the majority of participants described to have difficulties in detecting child maltreatment [17]. Uncertainty regarding potential maltreatment is known to be a major risk factor for inconsistent reporting to CPS by healthcare professionals. [18, 19].

In line with these findings, even if participants reported suspicion for maltreatment, only 80% chose to report to CPS. Even though 80 % seem to be a high percentage in a system without mandatory reporting, it has to be considered that known barriers for reporting, such as familiarity with the family [20], fear of negative consequences of a report for oneself, the patient or the family [19] and uncomfortability with addressing the issue of maltreatment with the family [21] may be a minor obstacle in a theoretical case vignette compared to real life.

Previous research suggests that another common reason for omitted reports to CPS might be a lack of feedback on the possible course of action when maltreatment is suspected. [18, 19]. In addition, many might shy away from reporting if they perceive CPS intervention as incompetent or insufficient—even if the legal norm warrants a report. But also lack of knowledge and experience seem to impact reporting. In our data, self-perceived good knowledge of the legal framework and work experience with cases of suspected maltreatment were positive predictors for reports to CPS.

Our data show that training in child protection is a predictor for the degree of perceived suspicion for child maltreatment in the vignette, but not for reporting the case to CPS. This is surprising and points towards a divergence of evaluation of the case (threshold for suspicion) and handling of the case (threshold for reporting). Consequently, child protection training should put more focus on the legal framework and subsequent implications for handling of the case beside recognition of child maltreatment.

Interestingly, work experience as proxy measured by participants’ age was not a predictor for higher level of suspicions or reporting to CPS. A cautious interpretation might be that one only sees what one is looking for, even as a healthcare professional: Many years of experience without cases of suspected maltreatment (whether not present or not detected) might add to a confirmation bias and making detection of and appropriate decisions harder, not easier.

The central limitation of the present study is that the participants cannot be considered as representative for their profession. Although almost all relevant German professional associations have supported the survey, the response rate of its members fluctuated and amounted to 3.9% overall participation. However, it should be noted that this response rate is based on the total number of members of the associations even though not all members received this call (as not all had agreed to receive emails by their association). Consequently, a higher actual response rate can be assumed. The gender ratio of the participants was almost exactly the same as that of the respective population, although on average they were younger (see Additional file 1). Another limitation is that questions on knowledge about child protection and the case vignettes were designed or adapted by the authors themselves - due to the lack of such instruments, psychometrically validated methods could not be used here. There are other factors besides personal suspicion of maltreatment and reasonable suspicion that are discussed to influence reporting. These include faith in the CPS, personal connection to the family, and decision-maker’s education [8]. While we have controlled for education, personal connection to the family cannot play a role in our case vignettes. As the latter usually keeps professionals from reporting, our results may even overestimate reporting rates.

## Conclusions

Taken together, this study is the first assessing the decision of healthcare professionals decision to “reasonably” report alleged incidents of child maltreatment in Germany. The results show that a significant proportion of health care professionals are uncertain about estimating reasonable suspicion and on how to proceed when there are strong indications for child maltreatment. Further training efforts seem to be necessary in order to improve reporting in behavior and consequently child protection in Germany.

## Supplementary Information

**Additional file1: Appendice 1.** Participating professional associations

## Data Availability

The datasets generated and/or analysed during the current study are not publicly available as this was not part of the informed consent form but are available from the corresponding author on reasonable request.
