# Supplementary material for: Reasonable suspicion in reporting child maltreatment: a survey among German healthcare professionals
Source: Child Adolesc Psychiatry Ment Health. 2021 Jun 14;15:28. doi: 10.1186/s13034-021-00381-7 (PMC8204433; doi:10.1186/s13034-021-00381-7)
Supplement: Supplementary file 1 — Additional file1: Appendice 1. Participating professional associations [file 13034_2021_381_MOESM1_ESM.docx]

**Appendice 1 Participating professional associations**

| **Associations** | **Registered Office** | **Participated in study** |
| --- | --- | --- |
| German Society of Pediatrics and Adolescent Medicine | Berlin | 1581 |
| German Society of Child and Adolescent Psychiatry. Psychotherapy and psychosoamtics | Berlin | 203 |
| German Society of paediatric surgery | Berlin | 58 |
| Bavarian Chamber of Psychotherapists | Munich | 47 |
| Chamber of Psychotherapists of Schleswig-Holstein | Kiel | 39 |
| Chamber of Psychotherapists Baden-Württemberg | Stuttgart | 202 |
| Chamber of Psychotherapists Lower Saxony | Hannover | 73 |
| Chamber of Psychotherapists Hamburg | Hamburg | 7 |
| Chamber of Psychotherapists of the Saarland | Saarbrücken | 16 |
| Chamber of Psychotherapists of Bremen | Bremen | 44 |
| Chamber of Psychotherapists of Berlin | Berlin | 13 |
| Chamber of Psychotherapists of Hesse | Wiesbaden | 37 |
|  |  |  |
| Chamber of Psychotherapists of North Rhine-Westphalia | Düsseldorf | 42 |
| East German Chamber of Psychotherapists | Leipzig | 118 |
| From Rhineland-Palatinate (Chamber of Psychotherapists did not participate in the study) |  | 5 |
|  |  |  |
| **Population** |  | **2481** |

Overall response rate: 3.92%

Mean age of participants in years: 44.8 (population: 50.8)

Gender of participants (women:men) in %) 69.69:29.87 (population: 70.95:29.04)
